# The Effect of Epidural Analgesia on Quality of Recovery (QoR) after Radical Prostatectomy

**DOI:** 10.3390/jpm13010051

**Published:** 2022-12-27

**Authors:** Ruben Kovač, Ivo Juginović, Nikola Delić, Ivan Velat, Hrvoje Vučemilović, Ivan Vuković, Verica Kozomara, Božidar Duplančić

**Affiliations:** 1 Department of Anesthesiology and Intensive Care, University Hospital Split, 21000 Split, Croatia; 2 Department of Urology, University Hospital Split, 21000 Split, Croatia

**Keywords:** quality of recovery, radical prostatectomy, general anesthesia, epidural anesthesia, postoperative analgesia, morphine, ropivacaine, tramadol, multimodal analgesia

## Abstract

No studies are currently regarding the quality of recovery (QoR) after open radical prostatectomy (ORP) and epidural morphine analgesia. This was a randomized, prospective, and controlled study that explored QoR on the first postoperative day after ORP. Sixty-one men were randomized into two groups. The first (epidural) group received general anesthesia combined with epidural anesthesia and postoperative epidural analgesia with morphine and ropivacaine. The second (control) group received general anesthesia and continuous postoperative intravenous analgesia with tramadol. Both groups received multimodal analgesia with metamizole. The primary outcome measure was the total QoR-40 score. Secondary outcome measures were: QoR-15, QoR-VAS and the visual analogue scale (VAS) for pain, anxiety and nausea. The median difference in the total QoR-40 score after 24 postoperative hours between the two groups of patients was 2 (95% CI: −3 to 8), *p* = 0.35. The global multivariate inference test for secondary outcomes between groups was not significant *p* > 0.05). QoR-VAS was correlated with QoR-40 (*r* = 0.69, *p* ≤ 0.001) and with QoR-15 (*r* = 0.65, *p* ≤ 0.001). The total QoR-40 and QoR-15 alpha coefficient with 95% CI was 0.88 (0.83-0.92) and 0.83 (0.77–0.89), respectively. There was no difference in the QoR between the epidural and the control group after ORP. The QoR-40 and QoR-15 showed good convergent validity and adequate reliability.

## 1. Introduction

Prostate cancer is the second most common cancer in men. It accounts for 13% of all newly diagnosed cancers. Approximately 12% of men will be diagnosed with prostate cancer during their lifetime [1]. Open radical prostatectomy (ORP) is the gold standard in surgical treatment for prostate cancer. The main goal of any surgical approach is cancer eradication, while preserving the pelvic organs’ function. Early postoperative quality of recovery (QoR) is an important outcome after ORP. These patients are older, they have cancer, and the surgery presents an intermediate risk. This study is investigating the early QoR after ORP using intraoperative and postoperative epidural anaesthesia and morphine compared with intravenous multimodal analgesia.

Although epidural morphine analgesia is used for long-lasting analgesia following a cesarean section [2,3,4], there are no studies regarding epidural morphine analgesia and radical retropubic prostatectomy. Contrary to local anesthetics, hydrophilic opioids such as morphine tend to remain in cerebrospinal fluid and produce longer analgesia—up to 24 h [5]. There are no studies regarding multimodal analgesia with intravenous tramadol and metamizole after ORP and their impact on QoR.

Tramadol is a weak agonist of mu opioid receptors. It also inhibits serotonin and norepinephrine reuptake, enhances inhibitory effects on pain transmission in the spinal cord, and therefore has analgesic properties; analgesic potency of tramadol is about 10% that of morphine [6]. Tramadol’s effectiveness is strongly impacted by a patient’s CYP2D6 function and can contribute to analgesic failure [7].

Postoperative QoR can be evaluated using psychometric questionnaires such as QoR-40 scale and QoR-15. The QoR-40 scale was developed by Myles et al. and it consists of 40 Likert scale questions; moreover, the summary score represents the total QoR.

The QoR-40 measures patients’ health status after surgery and anesthesia and it has been proposed as a measure of outcome in clinical trials [8].

The QoR-40 scale is extensively validated [9] and widely used in various clinical trials [10,11,12,13,14,15].

The primary objective of this study was to compare the effect of two anesthetic techniques, namely, general anesthesia followed by iv analgesia vs. general and epidural anesthesia followed by epidural analgesia, on the postoperative QoR-40 for patients undergoing radical prostatectomy. The secondary objective was to support the validity of the QoR-40 with instruments which evaluate QoR: QoR-VAS, QoR-15, and visual analogue scales for pain, anxiety, and nausea. The secondary outcomes were deemed to be associated with QoR-40.

## 2. Materials and Methods

This was a single centered, randomized, prospective, and controlled clinical trial. Examiners were blinded in the postoperative period. The study was approved by the ethics committee of the University Hospital of Split and the trial was registered under the number NCT04587505. All patients had been scheduled for elective radical retropubic prostatectomy between April 2019 and April 2021. Patients were excluded if they had contraindications for epidural anesthesia, dementia, intraoperative complications that required postoperative intensive care unit admission, Montreal Cognitive Test <24 points, or if they had refused to participate. Patients were informed about the study one day before the surgery and provided with informed consent. Participants were allocated by permuted block randomization into one of the two groups: the epidural group and the control group. The randomization list was obtained from R program version 3.5.3. with package Blockarand [16]. The group allocations were contained in closed envelopes that were opened before the surgery and after the enrolment procedure.

All participants received premedication with diazepam 5 mg p.o. 12 h and 1 h before the surgery. Thromboprophylaxis (low weight heparin 4000–6000 IU), depending on the body weight, was given at least 12 h before the surgery.

Patients were warmed to avoid unintended hypothermia. The induction of general anesthesia was performed with midazolam 2.5 mg, fentanyl 100 μg, propofol 1–2 mg/kg, and vecuronium 0.1 mg/kg. Balanced crystalloid fluids were used to treat hypovolemia. Additionally, 6% Hydroxyethyl starch was used before administering blood transfusion products to treat profound hypovolemia. Blood transfusions were given in cases of blood loss or other clinical indications. Bradycardia was treated with atropine. Hypotension was treated with ephedrine boluses. Neostigmine 2.5 mg with atropine 1 mg was used to reverse the effects of non-depolarizing neuromuscular blocking agents after surgery. Participants were placed in a urology high care unit for one day and were provided with constant and vigilant nurse care. Crystalloid infusions were used for maintaining diuresis. Pantoprazole 40 mg was used for gastroprotection. Metoclopramide 10 mg was administered for the prevention of postoperative nausea and vomiting (PONV).

The epidural group received general anesthesia and epidural anesthesia followed by postoperative epidural analgesia. Epidural catheter insertion was performed before the induction of general anesthesia using a midline approach at Th 12—L 1 level or Th 11–Th 12 level. A Safety check of the inserted epidural catheter was confirmed using lidocaine 60 mg. The epidural anesthesia included a mixture of ropivacaine 6.5 mg/mL and fentanyl 8.3 μg/mL. The general anesthesia was maintained with isoflurane in a mixture of 50/50 nitrous oxide and oxygen to achieve the minimum alveolar concentration between 0.6 and 0.8. The epidural loading dose was given according to our classification (3, 4, 5, or 6 mL) and titrated afterwards using the epidural boluses (1–2 mL). A loading dosage of 3 or 4 mL was carefully given to ASA III and elderly patients; 5 mL was given to ASA II and 6 mL to ASA I and young patients.

Before the end of the surgery, patients received 4 mL of the mixture of ropivacaine 4.4 mg/mL and morphine 0.8 mg/mL. Principal epidural analgesia was achieved with single shot morphine 3.2 mg.

Postoperative epidural analgesia was given in boluses for the following 24 h as a mixture of ropivacaine 2.2 mg/mL and morphine 0.4 mg/mL. Epidural analgesia with morphine and ropivacaine was administered by urologist in accordance with our classification based on morphine dosage as class I (2 × 0.8 mg), class II (2 × 1.2 mg), and class III (3 × 1.2 mg). Class I was given to ASA III and elderly patients, class II to ASA II, and class III to ASA I or young patients.

The control group consisted of patients who received balanced general anesthesia followed by postoperative iv analgesia with tramadol. The balanced general anesthesia was maintained by nitrous oxide and oxygen in a 50/50 mixture and isoflurane to achieve the minimum alveolar concentration between 0.8 and 1. Fentanyl was administered in a loading dose between 6 and 8 μg/kg. Additional fentanyl doses were administered incrementally, if needed. The dosage of postoperative iv analgesia with tramadol was 100 mg in the first hour, followed by 300 mg continuously during the following 24 h.

The anti-inflammatory drug metamizole (dipyrone) 2.5 g was administered intravenously before the end of the surgery and 12 h after the surgery (in both groups) to achieve multimodal analgesia.

Retropubic radical prostatectomy was performed in the Trendelenburg position. Median laparotomy was the preferred approach. Dorsal vascular complex was ligated to reduce blood loss and enable better visualization. The prostate was removed with care and the focus was to preserve the neurovascular bundle and the urethra, which are connected to the bladder neck to form the vesicourethral anastomosis. Extended lymph node dissection was performed when it was indicated by the risk factors. Two drains were placed inside the pelvic cavity and the wound was closed by interrupted sutures.

The primary outcome was a patient-rated visual quality of recovery-40 (QoR-40). The QoR-40 has five related dimensions of quality of recovery: emotional state (8 items), physical comfort (12 items), physical independence (5 items), psychological support (7 items), and pain (7 items). The summary score represents the total QoR. The minimal possible score is 40 and maximal possible score is 200.

A patient’s acceptable symptom state for the QoR-40 score is considered to be 180 points. The minimum clinically important difference (MCID) is the smallest change in an outcome that is considered relevant by patients [17]. If the difference in QoR-40 score is less than an MCID value of 6.3 points, then the difference in QoR-40 score is not important, regardless of statistical significance. Although the MCID indicates clinically relevant differences, it does not solve problems with data variability in the planning effect size and sample size’s calculation. Previous work has determined the mean and standard deviation of the QoR-40 score in a major surgery group to be 166 ± 15 [18]. This standard deviation is derived from a heterogenous surgical population. Our educated guess about variability in the sample size calculation was made following a study by Catro-Alves et al. [19]. We calculated that a sample size of 62 was needed for 10 points mean difference of the QoR-40 score with an alpha of 0.05 and power of 0.8.

QoR-15 is a shorter version of the QoR-40 questionnaire consisting of 15 items scaled from 0 to 10. The minimum score is 0, and the maximum 150 [20].

The QoR-VAS score was used as a criterium for validating QoR-40 and QoR-15. It is a patient-rating visual analogue scale whereby recovery is quantified by placing “X” on the line. The range of the line representing the score is from 0 to 100 millimetres. Poor recovery is represented on the left-hand side and is defined as: severe pain, nausea and vomiting, confusion, immobilization, eating difficulties, and problems with communication. Excellent recovery is represented on the right-hand side, defined as: no pain, comfort, alert, active, enjoying food, and communicating freely.

Visual analogue scales for pain during rest, pain during activity, and anxiety were evaluated 24 h after the surgery. It has been determined that a patient acceptable symptom state for VAS pain at rest is 33 points or less [21]. We consider a score of VAS pain on activity less than 40 points to be acceptable. It was proposed that a score of VAS anxiety greater than 34 indicates an anxious patient [22].

A VAS for nausea intensity estimated the worst nausea intensity during 24 h. We consider a VAS nausea score less than 30 points to be acceptable.

The purpose of the secondary outcomes was to test the convergent validity of the QoR-40 score. The convergent validity is a correlation between tests that measure the same or a similar construct. QoR-15 and QoR-40 share items, therefore correlation analysis will be inflated and not appropriate for testing convergent validity. We consider a VAS nausea score less than 30 points to be acceptable. The QoR-15 and QoR-40 share items, therefore correlation analysis will be inflated and not appropriate for testing convergent validity.

At the time of our study, there were no Croatian versions of the QoR instruments, therefore the authors translated QoR-40 and QoR-15 from English to Croatian language. A native English speaker performed back-translation. The simple content of the QoR instruments makes changes in semantics that might arise from the language’s translation; however, these changes are extremely unlikely to alter the instrument’s validity, and this fact has justified the use of the instrument in languages other than English despite the lack of formal validation [23,24]. We have tested the reliability and convergent validity of our Croatian version of QoR-40 and QoR-15. Correlation between QoR-VAS and QoR-40 and QoR-15 was considered to represent the convergent validity of the instruments. The reliability of the QoR-15 and QoR-40 was analysed using the Cronbach α test for internal consistency of items.

All data were analysed using descriptive statistics. The normality of the data was tested using the Shapiro–Wilk test and QQ plots. Fisher’s exact test was used for categorical variables. Equal variance was tested using the Levine test. Abnormally distributed data are displayed as median (interquartile range). Normal data are presented as mean ± standard deviation. Abnormally distributed data were tested using the Wilcoxon rank test and Spearman’s rank correlation test. Global multivariate inference test was used for nonparametric analysis of variance [25]. One test was used for QoR outcomes and one for VAS outcomes. The purpose of this robust test is to control a Type I error. A p-value less than 0.05 is considered to be statistically significant. Statistical analysis was performed using R program with RStudio. The R code is provided in Supplementary Materials.

## 3. Results

### 3.1. Participant Flow

**Figure 1:**
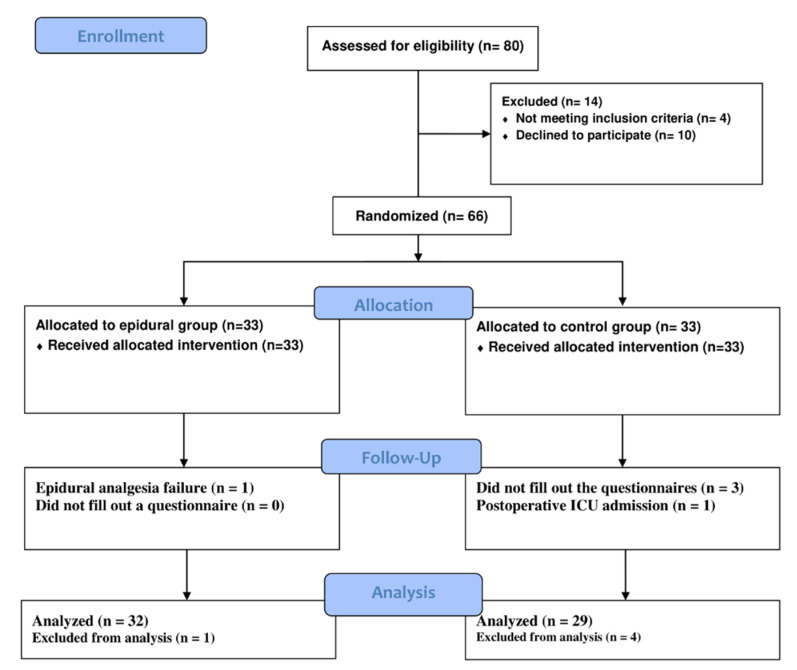
Flow diagram of the participants of the study.

From April 2019 until April 2021, 61 out of the 66 subjects originally randomized during that period completed the study (Figure 1). One of the limitations of our study is the fact that we did not achieve 31 subjects per group. We have managed to have a control group of 29 subjects due to the loss of 4 subjects in the follow-up. One of the patients in the control group had delirium, therefore he could not fill out the questionnaires coherently, and two patients refused to fill them out. One patient was admitted to the ICU because of prolonged surgery. In the epidural group, one patient had failure of epidural analgesia.

### 3.2. Descriptive Statistics

The mean age of patients was 67.3 ± 4.9 years. The mean surgery time was 184.5 ± 38.7 min. The QoR-40 median score was 181 (177–188).

The QoR-15 and QoR-VAS median scores were 124 (115–135) and 85 (76–90), respectively. Median score of the VAS pain during rest was 0 (0–20). The median score of the VAS pain during activity was 40 (20–40). The median scores of VAS anxiety and VAS nausea were 0 (0–0) and 0 (0–20), respectively. The VAS nausea score in our study was above 30 points in 22% of the patients. The outcomes were not normally distributed. Baseline data between the groups are presented in Table 1. In the epidural analgesia group, there was no patient in class III group. Q1 is defined as the middle number between the smallest number and the median of the data set.

### 3.3. Primary Outcome

**Figure 2:**
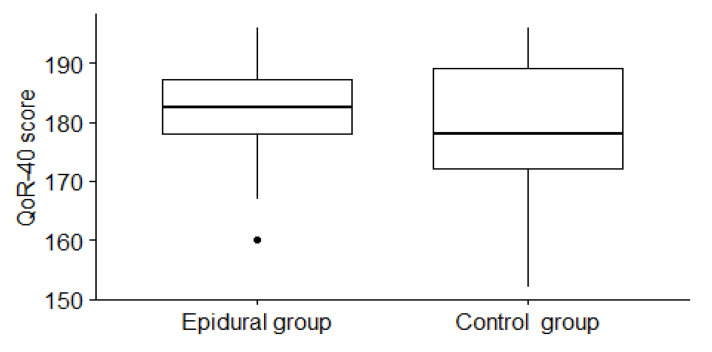
Global QoR: 40 scores in the epidural and control group.

The median difference in the global QoR-40 score during the first 24 postoperative hours between the epidural group and the control group was 2 (95% CI: −3 to 8), *p* = 0.35. The median score and IQR of QoR-40 during the first 24 postoperative hours in the epidural group was 182.5 (9.5) and, in the control group, was 178 (17) (see Figure 2). The Wilcoxon test can measure the effect size with Spearman r. The r value varies from 0 to 1. Despite the epidural group having a slightly better QoR-40 score, the Spearman r was 0.11. With an effect size of (*r* = 0.11), to obtain statistical significance, it is necessary to have 283 subjects per group.

Further analysis of dimensions has shown almost no difference in psychological support (*r* = 0.02) and emotional state (*r* = 0.01). Physical comfort, physical independence, and pain had r values of 0.12, 0.12, and 0.14, respectively.

### 3.4. Secondary Outcomes

All outcomes are present in Table 2. Global multivariate inference test for QoR outcomes (QoR-40, QoR-15, and OoR-VAS) was not significant (ANOVA type test *p* > 0.05). The QoR-15 effect size was small (*r* = 0.13).

**Figure 3:**
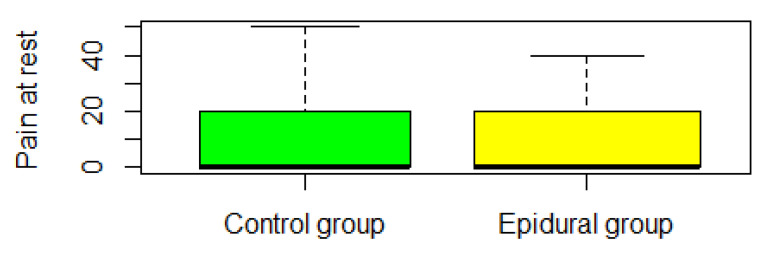
The VAS pain at rest score was obtained at the end of the analgesia protocol. This distribution is shifted toward zero, therefore the median is zero and the box is shorted.

**Figure 4:**
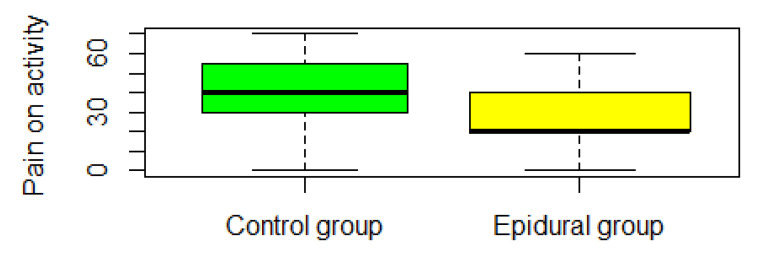
The VAS pain on activity was obtained at the end of the analgesia protocol.

**Figure 5:**
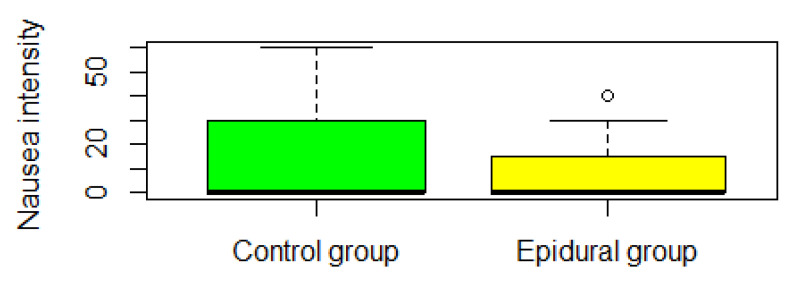
The VAS nausea intensity measured the worst patient score in the first postoperative day. This distribution is shifted toward zero, therefore the median is zero and the box is shorted.

Global multivariate inference test for VAS outcomes (pain on activity, pain at rest, and worst nausea in 24 h) was not significant (ANOVA type test *p* > 0.05) (see Figure 2, Figure 3, Figure 4 and Figure 5). VAS pain in rest score was significantly lower than 30 points in the one-sided Wilcoxon rank test, *p*-value = 1.655 × 10^−10^. VAS pain in the activity score was significantly lower than 40 points in the one-sided Wilcoxon rank test, *p*-value = 0.01.

QoR-VAS was strongly correlated with QoR-40 (*r* = 0.69, *p* ≤ 0.001) and with QoR-15 (*r* = 0.65, *p* ≤ 0.001).

Negative and significant correlation was found between total QoR instruments. VAS nausea intensity, pain during rest, pain during activity, and anxiety after 24 h (*p* ≤ 0.01) are presented in Table 3.

The QoR-40 and QoR-15 alpha coefficient with 95% CI was 0.88 (0.83–0.92) and 0.83 (0.77–0.89), respectively. Both alpha coefficients were above recommended values of 0.7.

## 4. Discussion

This study did not discover a statistically significant difference in the QoR-40 score between the two groups. The median score difference was less than an MCID value of 6.3 points. QoR-40 dimensions were consistent with the global QoR-40 score. The median QoR-40 score above 180 in this study suggests that patients had a good QoR.

Secondary outcomes: QoR-15, QoR-VAS, VAS pain at rest, VAS pain on activity, VAS anxiety, and VAS nausea did not differ between the groups. The QoR-15 median score difference was much smaller than an MCID value of 8. A median QoR-15 score above 118 in this study indicates a good QoR. The VAS pain scores were low at rest and on activity.

The correlation analysis demonstrated an existing association among outcomes that supports convergent validity.

We have decided to use QoR-40 as the primary outcome because it is the most comprehensive questionnaire. It is easy to understand and has 40 items relevant to QoR after anesthesia. It is the oldest instrument and is frequently used. We decided to use QoR-15 as a secondary outcome. The QoR-15 was made of the QoR-40 items with the most variance. Although QoR-15 contains only fifteen items, it consumes less time than QoR-40.

The VAS-QoR is a single-item instrument. There are no studies regarding this instrument. There are some psychometric problems with one-item instruments, such as lower or unknown reliability. However, the benefit of a one-item questionnaire is its application in time-restricted conditions and in vulnerable populations that have comorbidities which disturb their attention when completing longer questionnaires [26]. De Boder et al. successfully validated the single-item visual analogue scale (VAS), ranging from 0 to 100 for global quality of life [27].

There is no literature regarding ORP and QoR-40. A recent work by Beilstein et al. [28] evaluated analgesia after radical prostatectomy with QoR-15. They evaluated the effect of three different analgesic concepts: spinal single shot, TAP block, and the systemic administration of lidocaine. QoR-15 scores after 24 h did not differ significantly between the groups. One half of the patients had robotically assisted radical prostatectomy (RARP) and the other half had open radical prostatectomy (ORP). The pain intensity scores were similar after RARP and ORP. The numeric rating scale score during coughing was approximately 4 to 5, which is similar to VAS pain on activity in our study.

Konig et al. [29] compared postoperative pain scores between patients who were given intrathecal analgesia (morphine 300 µg or 240 µg for patients older than 70 years) with the patients who were not given intrathecal analgesia. The postoperative QoR-15 scores did not differ significantly between the groups; however, the relative decrease from the previous day was significant (10% vs. 13%). We did not measure the relative decrease (time and group interaction) because we wanted to analyze the intervention and its effect on both groups at the exact timeframe; it is not clear when the appropriate time for the measurement is, though. We decided to measure QoR in the first postoperative day because it was then that we expected to find most of the negative side effects of anesthesia and surgery. The discharge time could provide further information about patients’ early quality of life and QoR.

A study by Knipper et al. [30] showed similar postoperative pain intensity scores after RARP and ORP. Absolute QoR-15 values were similar; 123 points (106–137) vs. 118 points (105–130). Furthermore, they found similar postoperative analgesic requirements in RARP and ORP: metamizol: 11.5 g vs. 11 g and morphine equivalent: 28.3 mg vs. 30.0 mg. Those analgesia requirements are similar to our tramadol and metamizole protocol.

One of the limitations of our study is the fixed dosage of tramadol, which may have possible negative implications on QoR. Both tramadol and morphine epidural analgesia can be followed by nausea and vomiting. The postoperative PONV prophylaxis was less than is recommended [31], and this had clear implications on PONV and, consequently, on QoR-40 score. We did not measure pruritus, which can occur after morphine analgesia, although pruritus could have a negative effect on QoR.

The higher rate of dropouts in the control group could be due to a non-random effect if patients had very low QoR and could not proceed with questionnaires.

We were not able to blind subjects and examiners to group allocation; however, we made sure that the examiners who had collected QoR questionnaires in the postoperative period were not the same ones who had participated in anesthesia and postoperative analgesia.

This study did not perform a factorial design in order to differentiate the effect of anesthesia and postoperative analgesia on QoR. Two techniques of anesthesia and two techniques of analgesia could be combined into four combinations, such as, for example, general anesthesia and postoperative epidural analgesia or epidural anesthesia and postoperative intravenous analgesia. We did not consider factorial design because, in clinical work, we use combinations that have been presented in this study.

This study does not include prostatectomy specific outcomes such as sexual health, erectile function, emptying and storing capacity of the bladder, urinary obstruction and urinary incontinence, which can indicate the function and the quality of life [32].

This was a patient-centered study which should further inform clinical practice about two distinctive approaches of analgesia. There was no significant difference in the QoR-40 score between the groups. The analysis of the secondary outcomes indicates that there is no difference regarding QoR.

## Figures and Tables

**Table 1:** Baseline data between the groups. Data are presented as mean ± SD, *n* (%) or median (interquartile range). Data were analysed using independent t test, Mann–Whitney U test, or Fisher’s exact test.

Baseline Characteristic and Intraoperative Data				
		Epidural Group	Control Group	*p*-Value
		*n* = 32	*n* = 29	
Age (years)		68 ± 4.4	66.6 ± 5.5	0.367
ASA physical status (*n*)				
I		6 (18.8%)	7 (24.1%)	
II		22 (68.8%)	17 (58.6%)	0.73
III		4 (12.5%)	4 (13.7%)	
Surgery duration (minutes)		199 ± 39	167 ± 32	0.007
Anesthesia duration (minutes)		216 * ± 45	190 ± 42	0.07
Transfusion				
RBC	FFP ^+^			0.7
0 unit		25 (68.8%)	22 (75.8%)	
1 unit		2 (6.3%)	1 (3.4%)	
2 unit		5 (15.6%)	3 (10.3%)	
4 unit	+2 unit	0	1 (3.4%)	
8 unit	+4 unit	0	1 (3.4%)	
Crystalloids (mL)		1600 (500)	1500 (500)	0.19
Ephedrine (mg)		0 (5)	0 (10)	0.6
Atropine (mg)		0 (0.13)	0 (0.7)	0.5
Vecuronium (mg)		15.9 (2.5)	16.4 (3.7)	0.2
Fentanyl (mcg)		100 (0)	637 (161)	
Epidural analgesia		Class I (29%) Class II (71%) Class III (0%)		

* Includes time for placing epidural catheter. **^+^** Fresh frosen plasma (FFP) is given only together with red blood cells (RBC).

**Table 2:** Primary and secondary outcomes. Data are presented as median (IQR). Global multivariate inference test was used for obtaining *p*-value.

Primary and Secondary Outcomes			
	Epidural Group	Control Group	*p*-Value
	*n* = 32	*n* = 29	
QoR-40	182.5 (9.25)	178 (17)	*p* > 0.05
QoR-15	127 (18.25)	117 (20)	
QoR-VAS	84.5 (14.25)	85 (12)	
VAS pain at rest	0 (20)	0 (20)	*p* > 0.05
VAS pain on activity	35 (20)	40 (10)	
VAS nausea	0 (12.5)	0 (20)	
VAS anxiety	0 (0)	0 (0)	

**Table 3:** Correlational analysis of secondary outcomes.

VAS	Nausea Intensity	Pain at Rest	Pain on Activity	Anxiety
QoR-40	−0.40 *	−0.52 *	−0.62 *	−0.53 *
QoR-15	−0.48 *	−0.57 *	−0.63 *	−0.65 *
QoR-VAS	−0.42 *	−0.41 *	−0.50 *	−0.53 *

* *p* ≤ 0.01.

## Data Availability

The data presented in this study are available on request from the corresponding author. The data are not publicly available due to ethical restrictions.

## Supplementary Materials

The following supporting information can be downloaded at: https://www.mdpi.com/article/10.3390/jpm13010051/s1. The Supplementary Materials provide R code that was used for data analysis. R is a programming language for statistical computing and graphics. We hope that it will help others in future studies.
