# Supplementary material for: The Effect of Epidural Analgesia on Quality of Recovery (QoR) after Radical Prostatectomy
Source: J Pers Med. 2022 Dec 27;13(1):51. doi: 10.3390/jpm13010051 (PMC9862137; doi:10.3390/jpm13010051)
Supplement: Supplementary file 1 [file jpm-13-00051-s001.zip › jpm-2025119-supplementary.pdf]

R code

#Not all code here provided was used in data analysis but it can be used in similar research.

#the emphasis is on the method of data processing in r

```
#R packages
citation("npmv") # for citation
library(npmv)
library(tidyr) #for short to long data form
library("MPsychoR")
library("psych")
library("reshape2")
library(sjPlot)
library(PerformanceAnalytics)
library(corrplot)
library(MVN)
library(lavaan)
library(semPlot)
library(semTools)
library(tidyverse)
library(ggpubr)
library(rstatix)
library(DescTools)
library(coin)
library(ggplot2)
library(mvnormtest)
library(pwr)
library(effectsize)
```

The first problem with R for new beginners is how to import data in R enviroment.

```r
# in R studio, I have imported data saved in a very simple TXT file.

# I have to copy and paste all the variables from "Excel" or "Libre" table - without column name, just
#numbers. R studio is automatically naming it with V latter name: V1,V2,V3...

# It is important to know the names for renaming variables in R studio

# R studio - import dataset- from Text ...

#	Dealing with QoR-40 questioner:

# "Natural" order of items and dimensions in QoR-40

#Emotional state: 2,9,12,28,36,37,38,39,40

#Physical comfort: 1,5,10,11,19,20,21,24,25,26,27,34

#Psychological support: 13,14,15,16,17,18,35

#Physical independence: 3,4,6,7,8

#Pain: 22,23,29,30,31,32,33

# in our excel items are rearranged to follow the dimensions, not the natural order from QoR-40
questioner

# To create dimensions sum score we need to sum score of the items.

q$Comfort = as.numeric(apply(q[,1:12], 1, sum))

q$Emotions = as.numeric(apply(q[,13:21], 1, sum))

q$Independence= as.numeric(apply(q[,22:26], 1, sum))

q$PschyologicalSupport= as.numeric(apply(q[,27:33], 1, sum))

q$Pain= as.numeric(apply(q[,34:40], 1, sum))

#The sum of QoR score = global QoR score

# Creating the global score sum and name. First 40 columns are QoR-40 items, next 15 are QoR-15 items

q$QoR40= as.numeric(apply(q[,1:40], 1, sum))

q$QoR15= as.numeric(apply(q[,41:55], 1, sum)

#naming other variables

q$QoRVAS <- q$V56

q$Nausea <- q$V57

q$PainR<- q$V58

q$PainA<- q$V59
```

```
q$Anxiety<- q$V60

q$Epidural[q$V61 == '0'] <- 'Control    group' #changing name of binary variable outcome

q$Epidural[q$V61 == '1'] <- 'Epidural group'

q$subject<- factor(q$V62)

q$Age<- q$V63

q$ASA<- as.integer(q$V64) # ASA is ordinal variable.

q$Timeanest<- as.integer(q$V65)

q$Timesurgery<- as.integer(q$V66)

q$fentanyl<- factor(q$V67)

q$vecuronium<- as.integer(q$V68)

q$Epiduralloading<- factor(q$V69)

q$Analgesia[q$V70 == '0'] <- 'Control    group'      #renaming

q$Analgesia[q$V70 == '1'] <- 'Class I'

q$Analgesia[q$V70 == '2'] <- 'Class II'

q$cristaloid<- q$V71

q$RBC<- factor(q$V72)

q$FFP<- factor(q$V73)

q$atropin<- as.integer(q$V74)

q$efedrin<- q$V75

#data visualisation with boxplot      -    To avoid endless code we presented one example of code

boxplot(QoR40 ~ Epidural, data = q,

          xlab = " ",

          ylab = "QoR-40 ",

          notch = FALSE,

          varwidth = TRUE,

          col = c("green","yellow"),

          names = c("Control group","Epidural group")

)
```

```
#Normality analysis

describe(q)

# the problem with mvn test is that category variable needs to be excluded from analysis or test will not work.

# MVN test calculate univariate Shapiro-Wilk test, and descriptives.


m1<- mvn(data = q[76:86], mvnTest = c("mardia"), showOutliers = TRUE, univariateTest="SW")

r1 <- mvn(data = q[, c(76:85)],univariatePlot = "qqplot")

r2<- mvn(data = q[, c(76:85)], univariatePlot = "histogram")


#-qq plot analysis- To avoid endless code we presented one example of code

ggqqplot(q, x = "QoR40", facet.by = "Epidural")

ggqqplot(q, x = "QoR40")


#reliability calculation

QoR40= q[,1:40]

QoR15= q[,41:55]

tab_itemscale(QoR40,show.shapiro = TRUE, show.kurtosis = TRUE,use.viewer= TRUE)

psych::alpha(QoR40)          #Cronbach's =0.88,    (0.83 0.88 0.92)

tab_itemscale(QoR15,show.shapiro = TRUE, show.kurtosis = TRUE,use.viewer= TRUE)

psych::alpha(QoR15)          #Cronbach's =0.830; (0.77 0.83 0.89)


# descriptive statistics- to avoid endless code we presented one example of code

summary(q)

describe(QoRVAS ~ Epidural,IQR=TRUE, data=q)
```

nonparametric static -to avoid endless code we presented one example of code

```
wilcox.test( QoR40 ~ Epidural, data=q,conf.lev=0.95,conf.int=TRUE,exact=F,correct=T)

nonpartest(QoRVAS | QoR40 |QoR15    ~ Epidural, data = q, permreps = 10000)
# Permutation Test,Wilks Lambda 0.525
#                 QoRVAS   QoR40   QoR15
#Control group 0.50646 0.43965 0.41811
#Epidural group 0.49354 0.56035 0.58189

nonpartest( Nausea    |PainR |PainA | Anxiety ~ Epidural, data = q, permreps = 10000)
#Permutation Test p-value, #ANOVA type test p-value      0.45
#                  QoRVAS QoR40 QoR15
#control group 0.50646 0.4305 0.41811
#Epidural group 0.49354 0.5695 0.58189

#correlation analysis
mardia<- mvn(data = q[76:82], mvnTest = c("mardia"), showOutliers = TRUE, univariateTest="SW")
chart.Correlation(q[, c(76:82)], method= "spearman", histogram=TRUE, pch=19)

# QoR dimensions as predictors
nonpartest(Comfort| Emotions|PschyologicalSupport|Independence |Pain    ~ Epidural, data = q,
permreps = 10000)
#ANOVA type test p-value 0.39
```

```
# Wilcox R as effect size for nonparametric statistic- to avoid endless code we presented one example of code

q %>% wilcox_effsize(QoR40 ~ Epidural) # r= 0.12

#POWER ANALYSIS

#If effect size is 0.12, how much subjects is needed to get statistical significance, if #power=.8, and #alpha=0.05)

r_to_d(0.12) #0.2417469

#then we calculated in G power where cohen D is input-> 566 subjects is needed if r=0.12 (283 per group)

# Another calculation in R for Spearman r provided similar answer.

pwr.r.test(r=0.12, sig.level=0.05, power=0.80)

#n = 542; 271 per group



#Fisher's Exact Test for Count Data

table1 <- matrix(

  c(22, 1,

    3,1,

    1,25,

    2,5,

    0, 0),

  nrow = 2, byrow = TRUE,

  dimnames = list(

    "Treatment" = c("Control", "Epidural"),

    "RBC bags" = c("0", "1","2","4","8")

  )


fisher.test(table1)
```

```
#We did not used MANOVA or SEM, but maybe it can be used in other studies.

# MANOVA
outcome <- cbind(q$QoR40, q$QoR15, q$QoRVAS)
QoRModel <- manova(outcome ~ Epidural, data=q)
summary(QoRModel, intercept=TRUE)
summary.aov(QoRModel)

# STRUCTURAL EQUASION MODELING (SEM)
QoRmodel <- " QoR40 + QoR15 + QoRVAS ~ Epidural"
fit <- sem(QoRmodel, data = q)
summary(fit)
#This is model with no latent variable, just regression

#    Visualisation
semPaths(fit, "std", exoVar = TRUE, style = "lisrel", edge.color= "black" )

# Example of classical latent variable as predictor and indicators as outcome
#        ind60 =~ x1 + x2 + x3
```
